# Why does the magnitude of genotype‐by‐environment interaction vary?

**DOI:** 10.1002/ece3.4128

**Published:** 2018-05-08

**Authors:** Julia B. Saltz, Alison M. Bell, Jonathan Flint, Richard Gomulkiewicz, Kimberly A. Hughes, Jason Keagy

**Affiliations:** ^1^ Rice University Houston Texas; ^2^ University of Illinois at Urbana‐Champaign Urbana Illinois; ^3^ University of California Los Angeles Los Angeles California; ^4^ Washington State University Pullman Washington; ^5^ Florida State University Tallahassee Florida

**Keywords:** genetic variation, genotype‐by‐environment interaction, phenotypic plasticity

## Abstract

Genotype‐by‐environment interaction (G × E), that is, genetic variation in phenotypic plasticity, is a central concept in ecology and evolutionary biology. G×E has wide‐ranging implications for trait development and for understanding how organisms will respond to environmental change. Although G × E has been extensively documented, its presence and magnitude vary dramatically across populations and traits. Despite this, we still know little about why G × E is so evident in some traits and populations, but minimal or absent in others. To encourage synthetic research in this area, we review diverse hypotheses for the underlying biological causes of variation in G × E. We extract common themes from these hypotheses to develop a more synthetic understanding of variation in G × E and suggest some important next steps.

## 1 INTRODUCTION

**Figure 1:**
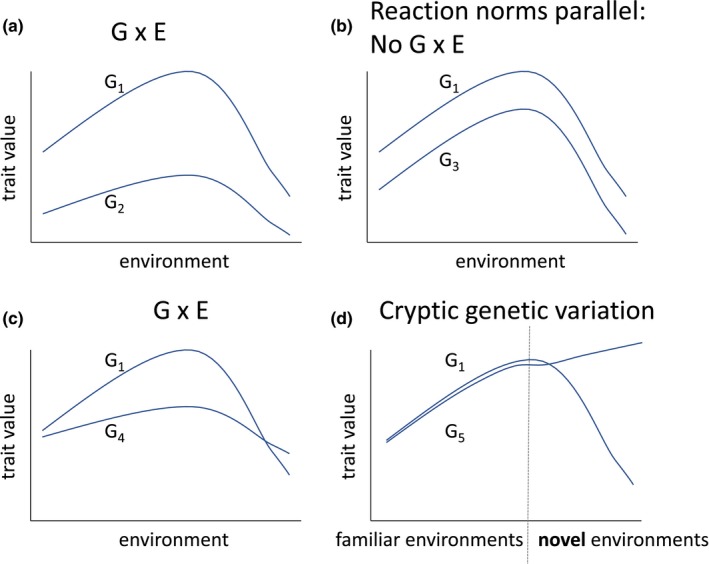
Examples of Genotype‐by‐environment interaction and its absence. Nonlinear reaction norms for hypothetical genotypes 1, 2, 3, 4, and 5. In panel a, the reaction norms of genotypes 1 and 2 do not cross but are not parallel over a range of environments: G × E. In panel b, the reaction norms of genotypes 1 and 3 are parallel over the entire range of environments studied: no G × E. In panel c, the reaction norms of genotypes 1 and 4 are not parallel over a range of environments, and cross: G × E. In panel d, reaction norms of genotypes 1 and 5 are similar and parallel across “typical” environments for their population, but diverge dramatically in evolutionarily novel environments, illustrating cryptic genetic variation

Genotype × environment interaction (hereafter “G×E”; sometimes abbreviated “GEI”) occurs when genotypes differ in the ways their trait values vary across environments (Figure 1). G × E is foundational to understanding the genetic basis of trait variation, with applications in genomics, evolutionary biology (Via & Lande, 1985, 1987), ecology (Miner, Sultan, Morgan, Padilla, & Relyea, 2005; Werner & Peacor, 2003), and human health. As the number of published studies reporting G × E grows, and our capacity to measure G × E at the molecular level becomes more precise and more available, a review and synthesis of hypotheses that may explain variation in G × E is timely.

As we review below, G × E can vary dramatically across traits and across populations. While variation in G × E presence and magnitude is discussed within individual studies, hypotheses explaining variation the presence and magnitude of G × E remain fragmented. Developing a more unified framework would be beneficial for illuminating the causes of trait variation and variation in trait plasticity, and how such variation may evolve. A predictive framework for G × E that integrates the effects of diverse mechanisms could be applied to critical health and conservation issues, such as predicting the vulnerability of populations to climate change, or predicting the effects of a particular drug given the patient's genotype.

To move toward such a framework, we review examples of variation in G × E estimates, describe hypotheses that could explain variation in G × E, and identify underlying themes and important future directions. Our focus is on the biological underpinnings of G × E and why they might differ across populations, species, or traits, generating G × E variation; we devote less attention to experimental design and statistical issues that might obscure true similarities or differences among G × E estimates (but see Box 1).

Box 1 **Methodological reasons for G × E variation**

1 In addition to the biological reasons for variation in G × E magnitude highlighted in the main text, differences in methodologies across studies may also produce different G × E estimates in different studies. Here, we highlight two of the most critical examples.

**Number and type of genotypes sampled**

Studies with more genotypes measured are more likely to include a genotype with an unusual reaction norm, producing larger G × E estimates than studies with fewer genotypes. The type of genotypes studied also matters. Replicate individuals with completely identical genotypes—such as clones or inbred lines—can be compared across environments, enhancing power to detect G × E (Falconer & Mackay, 1996). In studies of wild populations (or individuals sampled from the wild), genotypes are not fully replicated. Rather, the (typically unknown) loci responsible for G × E will occur on a variety of genetic backgrounds, obscuring G × E. Further, inbred strains derived from species that normally do not inbreed can be more sensitive to environmental effects than their wild (heterozygous) counterparts (Kristensen et al., 2005; Whitlock & Fowler, 1999), resulting in the potential for greater—but potentially less informative—G × E.

**Acclimation, habituation, and the timescale of experiments**

A change in the environment might evoke a strong immediate response that diminishes over time. Genetic differences in the rate at which such habituation or acclimation occurs could produce G × E: When individuals are compared for some trait before and after exposure to a novel environment, fast‐habituating genotypes should show relative aplasticity, while slow‐habituating genotypes, or genotypes that are sensitized by the novel environment, should show strong plastic responses. Variation in habituation and responses to novelty are well‐described in behavioral ecology (Brommer, 2013; Rodríguez‐Prieto, Martín, & Fernández‐Juricic, 2011); for example, the rate at which penguins habituate to the presence of humans depends on the penguin's age, sex, and personality type (Ellenberg, Mattern, & Seddon, 2009).

When genetic differences in habituation are present, even nearly identical studies that measure G × E over different timescales would be predicted to find different magnitudes of G × E. For example, in a study of G × E for gene expression in yeast, Eng et al. (2010) measured gene expression at 5, 15, 30, 45, 60, and 120 min after heat shock (Eng et al., 2010). For half the transcripts that showed G × E, G × E was detectable shortly after the heat shock but no longer detectable once the yeast had acclimated (Eng et al., 2010).

## 2 WHAT IS G × E?

G × E is estimated by measuring the trait values of different genotypes in different environments. Genotypes can represent any type of genetic grouping, such as clones, siblings, or populations. The environments might be discrete, such as light and dark; continuous, such as temperature; or random environmental effects, such as year. For each genotype, the array of trait values expressed across some set of environments is called the genotype's “norm of reaction” (Schmalhausen, 1949) or “reaction norm.” G × E is present whenever the reaction norms of at least two genotypes are not parallel (as in Figure 1a,c), that is, when genotypes differ in their trait values more in some environments than others (e.g., Figure 1a) or switch ranks in different environments (Figure 1c; Gupta & Lewontin, 1982), indicating genetic variation in plasticity.

## 3 EVIDENCE FOR VARIATION IN G × E

### 3.1 Direct comparisons of G × E for multiple traits in a single experiment

Perhaps the clearest signal of variation in G × E is when the same genotypes or individuals are measured for multiple traits across environments, and G × E estimates are compared across traits. For example, Valdar et al. (2006) measured 88 physiological and behavioral traits in different mouse genotypes (Valdar et al., 2006). In about half of traits exhibiting significant G × E, G × E explained >20% of variation, while in the other half G × E explained much less of the total variation, highlighting heterogeneity in the magnitude of G × E among traits. Further, over half of the G × E terms tested for physiological traits provided statistical support for G × E, but less than 5% of G × E terms for behavioral traits were statistically significant (although the effect sizes were similar), suggesting differences between trait categories (Valdar et al., 2006).

Similarly, many studies have investigated the quantitative genetics of gene expression. Here, the abundance of a particular mRNA transcript is considered a “trait” value. Like organismal traits, gene expression can be influenced by sequence variation, by the environment, and by G × E. By considering G × E for gene expression, thousands of transcript abundances can be measured from a single sample, providing direct, quantitative comparisons of G × E across traits. For example, several complementary studies of gene expression in yeast have revealed pervasive variation in G × E across traits and environments. In two studies, one that exposed yeast to different sugars (Smith & Kruglyak, 2008), and one that exposed yeast to heat shock (Eng, Kvitek, Keles, & Gasch, 2010), about 50% of transcripts showed evidence for G × E (Eng et al., 2010; Smith & Kruglyak, 2008). In contrast, two more yeast studies investigating still more environments (copper sulfate and different growth media) found G × E in less than 10% of transcripts studied (Fay, McCullough, Sniegowski, & Eisen, 2004; Landry, Oh, Hartl, & Cavalieri, 2006). These examples illustrate that different traits within the same individuals often differ in the extent to which they show G × E.

### 3.2 Variation in G × E among populations

Several studies have examined whether populations evolving in different types of environments show differences in the presence or magnitude of G × E. For example, Winterhalter and Mousseau (2007) studied G × E for diapause incidence within cricket populations from different latitudes, finding that some populations show significant G × E but other populations show no detectable G × E (Winterhalter & Mousseau, 2007). Similarly, McCairns and Bernatchez (2010) studied stickleback fish from freshwater and marine environments; by exposing fish to high‐ or low‐salinity environments, they found G × E for survival in freshwater, but not marine, populations (McCairns & Bernatchez, 2010). These examples illustrate how G × E often varies among different populations of the same species.

### 3.3 Population differences in reaction norms: The “ghost” of G × E past

Even when G × E is absent in a particular population—that is, all genotypes in that population show similar reaction norms—differences in (fixed) reaction norms *among* populations may reflect G × E that existed in the past. Many examples of such population differences in reaction norms have been documented (e.g., Murren et al., 2014; Oomen & Hutchings, 2015). These differences are expected to reflect different evolutionary histories of the two populations, that is, selection on the relevant trait values, and/or drift differed between the populations, resulting in the evolution of different reaction norms. These processes can produce differences between populations only when an ancestral population included genotypes with different reaction norms—that is, G × E. Thus, such population differences reflect historical G × E. However, variation in local adaptation—including whether the absence of local adaptation implies lack of G × E, or some other constraint—is still poorly understood (Moyle & Muir, 2010).

## 4 WHY DOES G × E VARY? UNDERLYING THEMES UNITING HYPOTHESES ABOUT G × E

**Figure 2:**
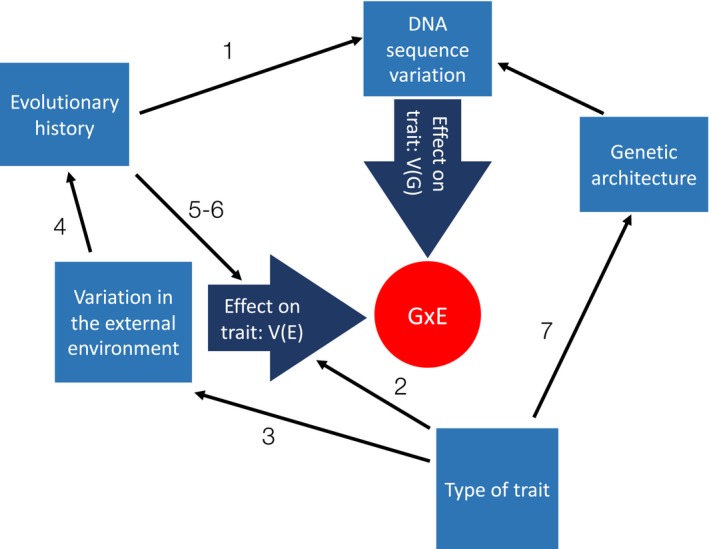
Conceptual flowchart illustrating processes expected to influence the magnitude of genotype‐by‐environment interaction (G × E). Variation in these processes across traits or populations is expected to produce concomitant variation in the magnitude of G × E. Specifically, we review hypotheses that:
1Populations or trait plasticities with greater genetic variation will have greater magnitude of G × E, relative to populations or trait plasticities with less genetic variation2More labile traits will show greater G × E than less‐labile traits3Genetic variation in niche‐constructing traits will generate G × E when exposure to environments influence reaction norms4Preference–performance correlations derive from and reinforce G × E5 and 6 G × E may be augmented or diminished when genotypes are studied in evolutionarily novel environments7Variation in plasticity due to large‐effect loci will result in greater magnitude G × E than variation in plasticity due to small‐effect loci

The above examples illustrate abundant variation in G × E both within and among populations and among traits. Many different, nonexclusive hypotheses for interpreting this variation are scattered across different literatures. Here, we first describe several unifying themes, and then articulate 7 hypotheses to explain variation in G × E (Figure 2).

Genotype‐by‐environment interaction describes genetic variation in trait plasticity; populations or traits lacking such variation will not show G × E. Thus, traits with “inherent” differences in *V*_G_ and *V*_E_ may also differ in the magnitude of G × E. Specifically, traits with greater genetic variation (*V*_G_) that are also more plastic (*V*_E_) are expected to show greater G × E than traits with low *V*_G_ and/or *V*_E_. *V*_G_ and *V*_E_ are technically independent parameters; but, empirically, across traits and organisms, estimates of *V*_G_ and *V*_E_ are positively correlated (Hansen, Pe, Houle, Pélabon, & Houle, 2011). For example, compared to morphological traits, behavior and life‐history traits exhibit higher additive genetic and also higher nonadditive and nongenetic variability (Hansen et al., 2011; Houle, 1992). Thus, highly variable traits are influenced both by substantial genetic variance and by substantial environmental (and nonadditive genetic) variance, providing greater purview for G × E.

For most traits, processes at the population level, and particularly a population's evolutionary history, is expected to shape the opportunity for G × E. Both neutral and adaptive processes “filter” which reaction norms remain in the population, but only across environments that the population has previously experienced. Furthermore, the types of environments experienced by a particular population, or even a particular genotype, can depend in part on individuals’ own behaviors, such as habitat choice (including “behaviors” in plants and other nonanimals, for example, germination cueing (Donohue, 2003, 2005). Such traits, referred to as “niche‐constructing” traits, influence the environments that individuals experience, thus uniquely *enabling* G × E over both short and long evolutionary timescales. At the same time, niche‐constructing traits are themselves subject to G × E.

This apparent complexity in the relationships between *V*_G_ and *V*_E_ indicates the need to identify the underlying causes of G × E. In quantitative genetics models, G × E is “just” a statistical parameter to be estimated; it does not provide any information about *how* or *why* genotypes vary in trait plasticity across measured environments (Bell & Dochtermann, 2015). While this level of generality is helpful for comparing across diverse environments, species or traits, it is less helpful in understanding why G × E varies and in forming predictions about G × E in new situations. A more mechanistic understanding of G × E at the organismal, functional, and molecular levels would help explain variation across traits in genetic architecture, functional similarities between genetic and environmental perturbations, and what allelic effects may be “exposed” in novel environments.

### 4.1 Hypothesis 1: Magnitude of *V*_G_: Populations or reaction norms with greater genetic variation will have greater G × E, relative to those with less genetic variation

Because G × E requires genetic variation, reaction norms with substantial underlying genetic variance should show greater G × E than reaction norms with low genetic variance. What, then, predicts the degree of segregating variation in reaction norms?

There is a large literature exploring the factors generating and maintaining genetic variation in traits within a single environment. Many of these ideas have been extended to consider genetic variation in trait *plasticity* across environments, that is, G × E. For example, effective population size is a critical determinant of neutral and adaptive processes affecting the magnitude of V_G_, and thus the magnitude of G × E. Selection is more efficient in larger populations, indicating that when populations evolve under similar, strong, directional selection on reaction norms, larger populations are expected to show a lower magnitude of G × E than smaller populations. When selection is weak, the opposite trend is expected: larger populations have more individuals, and thus, larger populations are more likely to harbor genotypes with unusual reaction norms, resulting in greater G × E than expected in smaller populations (Falconer & Mackay, 1996).

Other population‐genetic processes that add or remove genetic variation should also increase or decrease the magnitude of G × E observed, respectively. One such process is gene flow: In the study of diapause incidence in crickets (introduced above), populations at lower latitudes, where average plasticity was low and expected to be maladaptive, had greater G × E than populations at higher latitudes (Winterhalter & Mousseau, 2007). The authors suggest that G × E was maintained by gene flow from higher latitudes. Similarly, populations with greater mutation rates should show greater G × E than populations with lower mutation rates. Moreover, environments themselves may differ in how mutagenic they are (Bjedov et al., 2003; Visscher et al., 2004) indicating that environments can influence both selection for plasticity and the opportunity for G × E.

Selection may also influence the magnitude of G × E. As noted above, strong directional or stabilizing selection on reaction norms is expected to deplete genetic variation in plasticity, removing G × E. In this case, the trait may be plastic, but all genotypes will be plastic in the same way. Further, under long‐term stabilizing selection, phenotypes that initially show plasticity may evolve greater “resistance” to environmental effects and become canalized (Crispo, 2007; Waddington, 1942; West‐Eberhard, 2003). Thus, traits under strong stabilizing selection, such as those essential for viability, may be more canalized and thus have a limited scope for G × E, compared to other traits. Consistent with this idea, a study of G × E for gene expression in yeast found that essential genes (those causing lethality when deleted), were less likely to show G × E than nonessential genes (Landry et al., 2006). Together, these factors predict that populations evolving under stronger, more directional selection on reaction norms should show less G × E than populations with weaker, nondirectional selection.

In contrast, other forms of selection on reaction norms (e.g., disruptive selection, negative frequency‐dependent selection) may adaptively preserve intrapopulation genetic variation in reaction norms (Hedrick, 2006; Turelli & Barton, 2004). Indeed, G × E itself is often postulated as a mechanism of nonlinear selection by which trait variation may be maintained. However, it is G × E in fitness, rather than G × E for any particular trait, that can result in the adaptive maintenance of variation. Indeed, G × E at the level of fitness can be manifest without G × E in any of the component traits (Génard, Lescourret, Bevacqua, & Boivin, 2017). The critical requirement for the adaptive maintenance of G × E is the absence of any segregating genotype that is favored in all environments; if such a genotype existed, it would sweep to fixation, producing a monomorphic population. Therefore, G × E can be adaptively maintained when selection is heterogeneous, and when constraints (genetic, physiological, or other constraints) prevent any one genotype from producing the optimum phenotype in all relevant environments.

### 4.2 Hypothesis 2: Magnitude of *V*_E_: More labile traits will show greater G × E than less‐labile traits

Just as traits (or populations) with greater *V*_G_ should, in turn, show more G × E, traits with high environmental variance, reflecting plasticity, should show greater G × E than traits that are relatively aplastic. The magnitude of *V*_E_ is shaped in part by evolution: Classic work has shown that, even when populations experience the same range of environments, differences in how these environments are arranged in space and time can profoundly affect the evolution of plasticity (Via & Lande, 1987).

In addition, *V*_E_ can vary across “types” of traits. For example, in the mouse study described above (Valdar et al., 2006), physiological traits had substantially higher levels of common‐environmental variance than behavioral phenotypes, and concomitant greater magnitude of G × E. Similarly, meta‐analyses have demonstrated that behavioral and life‐history traits typically have greater magnitude of additive genetic variation (*V*_A_, the additive component of *V*_G_), *and* residual variation (which includes *V*_E_), relative to morphological traits (Hansen et al., 2011; Houle, 1992), suggesting substantial purview for G × E (Figure 2).

### 4.3 Hypothesis 3: Genetic variation in niche‐constructing traits will generate G × E when exposure to environments influence reaction norms

**Figure 3:**
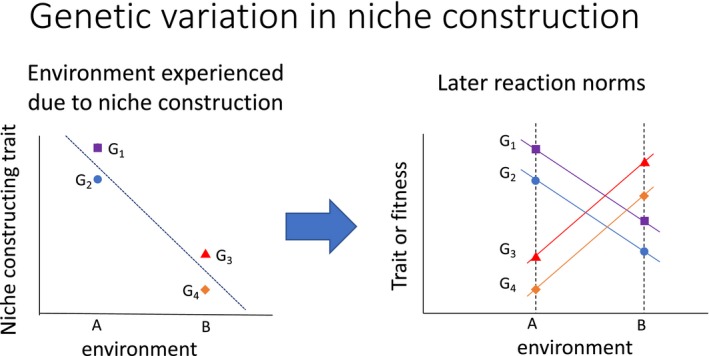
How genetic variation in niche construction may produce G × E. Individuals’ experience in particular environments, which is a function of their niche‐constructing traits (left) may influence their reaction norms when measured at a later time (right) resulting in G × E. Left: Genotypes 1 and 2 have high levels of a niche‐constructing behavior (e.g., sociability) and therefore occur in environment a (e.g., large group size), while genotypes 3 and 4 have low levels of the niche‐constructing behavior and therefore occur in environment b. Due to experiences in environments a or b, genotypes develop differences in their reaction norms across environments a and b (right), resulting in G × E. In this example, genotypes have higher trait (or fitness) values in the environment (a or b) they previously experienced, relative to the alternate environment, but other patterns are possible

Central to the idea of G × E is that individuals with different genotypes are measured across the same range of environments. However, in nature (and, arguably, even individuals under “controlled” laboratory conditions; Box 2), individuals have the opportunity to choose and manipulate their own environments, that is, niche construction (Odling‐Smee, Laland, & Feldman, 1996). When genotypes differ in niche‐constructing traits—for example, when some genotypes choose one environment, and other genotypes choose another—we expect different genotypes to systematically experience different environments (Figure 3, left; Eaves, Last, Martin, & Jinks, 1977; Plomin, DeFries, & Loehlin, 1977; Saltz, 2011; Saltz & Nuzhdin, 2014). For example, different cottonwood (*Populus*) genotypes differ in the concentration of tannins in their leaves, altering the chemical composition of the soil below them where the leaves decompose (Driebe & Whitham, 2000; Whitham et al., 2003). In *Drosophila melanogaster*, social groups differing only in the genotype of a single male have different group dynamics (Saltz, 2013, 2017; Saltz, Geiger, Anderson, Johnson, & Marren, 2016). Siblings with different personalities may receive different parental care, even from the same parents (Hayden et al., 2009; Plomin et al., 1977).

Box 2 **Can environments actually be “controlled”? Niche construction and the mismeasure of environments**

1 In addition to influencing G × E directly (hypotheses 3–4), genetic variation in niche construction can confound attempts to measure G × E. Specifically, genetic variation in niche construction implies that external measures of available environments may not reflect any individual's actual experiences. For example, researchers often assume that individuals who travel over greater distances experience a larger range of environments, relative to individuals who remain within a smaller spatial area (Mery, Belay, So, Sokolowski, & Kawecki, 2007; Sih & Del Giudice, 2012). This assumption ignores choices made by individuals during movement. Snell‐Rood and Steck (2015) measured the movement behaviors of butterfly genotypes that were either adept at dispersing long distances (i.e., genotypes with larger thoraces and more‐elongate wings), or not (Snell‐Rood & Steck, 2015). Unexpectedly, the genotypes expected to move greater distances explored their environments less thoroughly, honing in on a relatively narrow range of host types, while the less dispersive genotypes experienced a broader range of host types (Snell‐Rood & Steck, 2015).

When estimates of which individuals experienced which environments are biased by genetic variation in niche construction, estimates of reaction norms for each genotype are correspondingly biased. As a hypothetical example, highly dispersive genotypes might be assumed to be relatively aplastic if they maintain similar trait values across what appears to be a large range of environmental conditions, when in fact they may maintain consistent trait values because they choose to experience only a narrow range of environments. This phenomenon could explain the intriguing finding that the repeatability of behavior is higher under field conditions than under laboratory conditions (Bell, Hankison, & Laskowski, 2009).

These examples suggest that even in the “same” environments, different genotypes may have different experiences (Plomin et al., 1977; Saltz & Nuzhdin, 2014). One obvious method to overcome these limitations is to reproduce the relevant environments in the laboratory and measure the traits of different genotypes there. However, even laboratory environments may not be fully controllable. For example, playback experiments in birds can provide a controlled way of simulating social interactions, such as territorial intrusions (Nowicki et al., 2002); but even this approach is imperfect for detecting and interpreting individual variation (McGregor, 2000). In addition, genetically identical fish reared under seemingly identical environments in the laboratory can still develop highly repeatable, radically different behaviors (Bierbach, Laskowski, & Wolf, 2017).

In general, one of the most important steps for investigating G × E when genotypes vary in niche‐constructing traits is to simply acknowledge this possibility. Directly measuring the experiences of each individual, rather than assuming that all individuals in the same experimental treatment experience the same environment, can substantially improve our interpretation of experiments designed to measure G × E (Saltz, 2017).

Genetic variation in niche construction has a number of implications for studying G × E. First, genetic variation in niche construction can confound attempts to measure G × E (see Box 2). Second, genetic variation in niche construction may cause G × E when individuals’ environments influence their later reaction norms. Many experiences that individuals have in their environments, such as exposure to stimuli, social experience, and exposure to trauma, can have long‐lasting effects on trait plasticity (reviewed in Stamps, 2016). If different genotypes have different experiences, and different experiences lead to the development of different reaction norms at some later time (i.e., when individuals are measured), then G × E would be observed (Figure 3).

In cases where genetic variation in niche construction is the underlying cause of G × E, variation in G × E can (in theory) arise due to differences in whether genetic variation in niche construction is expressed. If the potential for genetic variation in niche construction is abrogated—for example, if preferred environments become unavailable (or are made unavailable by experimenters; Box 2), or if individuals are measured when they are too young to engage in niche construction—the magnitude of G × E should be diminished, relative to situations in which genetic variation in niche construction is expressed. Furthermore, if genetic variation in niche construction is an important contributor to G × E, then populations lacking variation in niche‐constructing traits should have lower‐magnitude G × E relative to populations with substantial genetic variation in niche construction.

### 4.4 Hypothesis 4: Preference–performance correlations derive from and reinforce G × E

One of the most common predictions about genetic variation in niche construction is that genotypes should be selected to choose the environment that is “best for them,” that is, maximizes their fitness. This process should result in a genetic correlation between preference for a particular environment type, and performance (fitness) in that environment (a “preference–performance correlation”; Gripenberg, Mayhew, Parnell, & Roslin, 2010).

Preference–performance correlations fundamentally rely on G × E, because it is only adaptive for genotypes to choose a particular environment if they have lower fitness in other environments. (One caveat is that G × E may exist at the level of fitness but not be evident in any individual traits (Génard et al., 2017).) For example, if genotype A has high fitness in environment A and low fitness in environment B, it should choose A. If genotype B has high fitness in environment B and low fitness in environment A, it should choose B. When these genotypes are measured in both environments, G × E will be observed. If a genotype has equal fitness in all environments, it would not be selected to have any habitat preference, and no preference–performance correlation or G × E would result.

At the same time, if genotypes are able to systematically avoid habitats in which they have low fitness, then reaction norms including low fitness in most environments can persist in the population. Therefore, populations in which preference–performance correlations are strong should harbor more G × E than populations lacking preference–performance correlations.

### 4.5 Hypothesis 5: The magnitude of G × E should be greater when environments studied include both “familiar” and “novel” environments

Just as genotypes may have altered trait values in environments that they avoid (Hypothesis 4), populations may have unpredictable reaction norms in novel environments, that is, those which have not been experienced by a population in its recent evolutionary history. Novel environments can disrupt physiological functions such as homeostasis, leading to developmental breakdown (“environmental stress”; Badyaev, 2009; Ghalambor, McKay, Carroll, & Reznick, 2007), which in turn may allow the expression of genetic variants whose phenotypic effects are normally suppressed. Such “cryptic” genetic variants have never been exposed to selection, and thus should influence trait values in random directions, including maladaptive directions (Ghalambor et al., 2007, 2015). A complementary set of hypotheses, focused on developmental behavioral plasticity at the organismal level, suggests that organisms may respond unpredictably in environments about which they have no information, that is, novel environments (Stamps & Frankenhuis, 2016). Thus, genotypes may show relatively canalized reaction norms when measured across a range of familiar environments, but the same genotypes may differ dramatically in their responses to novel environments, producing G × E (Figure 1d).

Cryptic genetic variation is expected to produce variation in G × E magnitude when populations are compared across environments that are novel for some populations but familiar to others. In this case, the populations experiencing (their) familiar environments are expected to show lower‐magnitude G × E than populations experiencing novel (to them) environments.

### 4.6 Hypothesis 6: Environments might be “too novel”: variation in sensitivity to stimuli should mediate the opportunity for G × E

Plasticity occurs when individuals sense stimuli in the environment and then respond by adjusting some aspect of their phenotype. G × E, then, arises due to differences among genotypes in “sensitivity”—that is, the ability to perceive variation among stimuli of different types or strengths—and/or in “responsiveness”—that is, how information they perceive is transformed into phenotypic change (or not). Thus, in contrast to hypothesis 5, novel environments might produce lower G × E estimates when stimuli in the environment are so novel that organisms fail to perceive them, curtailing both plasticity and G × E. For example, some individual birds might be better than others at hearing a song playback (McFarlane, Söderberg, Wheatcroft, & Qvarnström, 2016), and individual birds also vary in how strongly they react to the playback (Nowicki, Searcy, Krueger, & Hughes, 2002). Thus, a bird may fail to change its behavior following a song playback because the bird failed to perceive the song, or because the bird perceived the song but ignored it.

Understanding sensitivity and responsiveness as components of G × E may be beneficial for interpreting and predicting variation in G × E in novel environments. For example, native organisms may fail to respond to invasive predators, resulting in endangerment of native species (Blumstein, 2006; Sih, Trimmer, & Ehlman, 2016). Even within a population's familiar environments, populations lacking sensitivity to a particular stimulus should be unable to evolve responsiveness to that stimulus, inhibiting the potential for G × E. Further, more sensitive genotypes might show greater phenotypic plasticity for multiple phenotypes, producing a positive genetic correlation between different measures of plasticity (Saltz, Hessel, & Kelly, 2017; Saltz, Lymer, Gabrielian, & Nuzhdin, 2017).

### 4.7 Hypothesis 7: Genetic architecture: Variation in reaction norms due to large‐effect loci will result in greater G × E than variation in reaction norms due to small‐effect loci

The number and effect size of loci underlying trait plasticity should affect the likelihood of observing G × E, and its magnitude. Trait plasticities with a larger underlying mutational target size should be more likely to harbor variation in one or more functionally relevant loci, causing G × E, relative to trait plasticities with relatively small mutational target sizes. When the plasticities of two different traits are produced by similar number of loci, the effect sizes of those loci should influence which trait exhibits greater G × E; large‐effect variants that cause trait plasticities should produce relatively large‐magnitude G × E, relative to plasticities produced by a similar number of small‐effect variants.

These relatively simple predictions are substantially complicated by our ignorance about the types of genes that influence plasticity and the factors determining their effect sizes. Genes might influence plasticity in one or more organismal phenotypes either by changing in expression or function across environments (sometimes termed “allelic sensitivity”), or by exerting phenotypic effects in one environment but not another (Schlichting & Pigliucci, 1995). One study of plasticity in cichlid jaw morphology in response to hard‐ or soft‐food diets found both types of patterns: Some candidate genes for jaw plasticity showed high expression under one diet type, but very low expression under the other diet type, other candidate genes showed moderate, but different, expression under both diets, and still others showed similar expression across diets (Schneider, Li, Meyer, & Gunter, 2014). Further, these patterns depended on the developmental stage in which expression was measured.

What might influence the relative effect sizes of variants in such genes? In theory, for a given trait, variants in genes that regulate many downstream targets—such as transcription factors—are expected to have large effects on the resulting trait values, whereas downstream genes may have smaller effects (Marjoram, Zubair, & Nuzhdin, 2014; Nuzhdin et al., 2009). Thus, populations harboring variation in upstream “master regulators” of trait plasticity should show greater G × E than populations harboring variation only in downstream targets. Indeed, in the cichlid study described above, the candidate genes studied had binding sites for the same transcription factor—a transcription factor affected by the mechanical strain induced by a diet of hard foods (or lacking in a diet of soft foods; Schneider et al., 2014). However, the structure of genetic networks—that is, whether a particular gene is a “master regulator” or not—can depend on variants at other loci (i.e., epistasis; Chandler, Chari, & Dworkin, 2013; Chandler, Chari, Tack, & Dworkin, 2014; van Swinderen & Greenspan, 2005) and can vary across environments (Chiang et al., 2012). Furthermore, the evolutionary forces shaping these effect sizes may depend on whether a particular locus is subject to epistasis and/or has effects on multiple traits.

Overall, hypotheses linking variation in the genetic architecture of phenotypic plasticity to variation in G × E estimates are currently difficult to evaluate directly, because the allelic basis of G × E is typically unknown (Box 3).

Box 3 **The elusive allelic basis of G × E**

1 Although G × E must result from differential effects of specific alleles across relevant environments, attempts to identify interactions between environments and specific variants within genes have been largely unsuccessful (Duncan & Keller, 2011; Keller, 2014; Munafò, Durrant, Lewis, & Flint, 2009). One of the most important findings to emerge from genome‐wide association studies (GWAS) is that an individual locus makes only a tiny contribution to phenotypic variance: A typical locus explains far less than 1% of variation in trait values (Purcell et al., 2009; Rockman, 2012; Sullivan, Daly, & O'Donovan, 2012). Sample sizes needed to detect these loci in a GWAS design number tens of thousands; for finding a locus‐by‐environment interaction, even larger numbers are needed (Smith & Day, 1984).

At the transcript level, investigators have the opposite problem: thousands of transcripts and gene regulation mechanisms change across environments in ways correlated with phenotypic reaction norms (e.g., Ben‐Shahar, Robichon, Sokolowski, & Robinson, 2002; Harris & Hofmann, 2014); but connecting gene expression (and other molecular) variation to reaction norms at the organismal level is challenging (Bell & Dochtermann, 2015). Furthermore, interpreting the biological meaning of gene expression networks can be difficult in the absence of a priori null hypotheses (Sorrells & Johnson, 2015).

One promising solution for identifying causal alleles, particularly relevant to model organisms, is to increase the frequency of alleles relevant to plasticity by artificially selecting for particular reaction norms, for example, for particular learning abilities (Dunlap & Stephens, 2009; Mery & Kawecki, 2004), responses to conspecifics (Edwards, Rollmann, Morgan, & Mackay, 2006; van Oortmerssen & Bakker, 1981), or the ability to survive in multiple environments (Friesen, Saxer, Travisano, & Doebeli, 2004). Genome sequences of evolved and control populations can then be compared to identify alleles potentially causing differences in reaction norms (Turner & Miller, 2012). To further augment power to detect causal loci, DNA sequence information can be combined with information about how genes change their expression and functions across environments, using methods such as transgenics, chromatin availability, epigenetic modifications, and the presence of binding sites for particular transcription factors (Ayroles et al., 2009; Marjoram et al., 2014; Peng, Hassan Samee, & Sinha, 2015). Implementing these approaches is nontrivial, but promises to enhance our ability to hone in on causal loci and ultimately identify the molecular basis of G × E (Bell & Dochtermann, 2015).

## 5 WHAT WE STILL DO NOT KNOW: MOVING TOWARD A PREDICTIVE FRAMEWORK OF G × E

Considering hypotheses 1–7 together is a step toward a unified predictive framework of G × E, and raises several questions for future work.

First, most of the hypotheses proposed above are mutually compatible, that is, all of the relevant processes could be acting simultaneously on a given population or trait to produce the G × E estimate observed. What is the relative contribution of these different mechanisms to resulting variation in G × E magnitude? Quantitative geneticists are accustomed to decomposing variation in observed trait values; decomposing variation in population‐level parameters, that is, G × E, will prove more challenging. A starting point is for G × E researchers to begin testing multiple hypotheses (those above, or new ones) in a single experiment. For example, investigators could test for cryptic genetic variation (hypothesis 5: more G × E in novel environments) in both highly labile and less‐labile traits (hypothesis 2: more G × E in more labile traits), or for the effects of genetic variation in niche construction (hypotheses 3–4: genetic variation in niche construction can produce and/or magnify G × E) across population sizes (hypothesis 1: larger populations should have more G × E).

A second key question concerns the functional and molecular basis of G × E. Which types of genes and mutations are most likely to produce G × E? What determines variation in the genetic architecture of G × E? Our ability to measure G × E at the molecular level is at an all‐time high, but answers to these questions are still lacking. For example, “toolkit genes,” that is, genes that are repeatedly recruited for similar functions in different organisms, may be especially likely to contribute to G × E because they have important organismal functions (Carroll, 2008); or they may be less likely to contribute to G × E, relative to nontoolkit genes, because they are essential for viability and thus unlikely to vary substantially within natural populations (Landry et al., 2006). Finally, the alleles underlying G × E might not directly “encode” trait plasticity, but rather act to influence the environments that individuals experience, thereby producing G × E “indirectly” (hypothesis 3; Saltz & Nuzhdin, 2014).

Of course, our ability to measure environments is also at an all‐time high, but important questions remain about what types of environments evoke plasticity and G × E. Novel environments, in particular, are predicted to produce particularly strong G × E (hypothesis 5) or no G × E at all (hypothesis 6). Predicting G × E in novel environments is becoming increasingly important as climate change accelerates. Furthermore, “environments” are actually collections of diverse stimuli and experiences, which may have concomitantly diverse impacts on individuals’ trait values (Moffitt, Caspi, Rutter, Centre, & Kingdom, 2006). Thus, even when important environments are known, the relevant stimuli and the mechanisms by which they induce plasticity are usually not.

Progress on these relatively novel questions will ultimately illuminate a classic, fundamental question about G × E: what prevents any single genotype from having “perfect plasticity,” that is, optimal trait values in all environments? Investigating the causes of G × E at the molecular, organismal, and population levels will contribute to our understanding of the tradeoffs, costs, and/or limits that enable the evolutionary maintenance of G × E.

## 6 CONCLUSIONS

Genotype‐by‐environment interaction exists at the nexus of ecology and evolution: It describes how the expression of genetic variation is modified by the environment. Although G × E has been extensively documented, many questions remain about what factors determine its magnitude. Developing a predictive framework that explains variation in G × E is essential for understanding how trait variation arises. Here, we have highlighted a nonexhaustive list of nonexclusive mechanisms that are expected to systematically produce variable G × E estimates across studies, organisms, and traits. Our goal is to encourage G × E researchers to move beyond quantifying G × E and to begin to quantitatively test predictions about how and why G × E varies.

## 7 OUTSTANDING QUESTIONS ABOUT VARIATION IN G × E

- Which types of genes and mutations are most likely to produce G × E? Can we predict G × E from genetic information alone?
- How does G × E at the level of gene expression translate to G × E for organismal traits?
- Is G × E typically greater or smaller in evolutionarily novel environments?
- Are there generalities about the selective conditions, including ecology and life histories, that promote G × E? For instance, do we expect more or less G × E for organisms that disperse, undergo diapause, have seed banks?
- How do we expect the opportunity for G × E to change over the lifecourse?
- Are we more or less likely to detect G × E for “toolkit genes,” that is, genes that are repeatedly recruited for similar functions in different organisms?
- How can more computationally sophisticated modeling approaches that integrate multiple types of genomic data (ChIP, SNP, gene expression) be developed to discover the molecular basis of G × E?

## CONFLICT OF INTEREST

The authors declare no competing financial interests.

## AUTHOR CONTRIBUTIONS

All authors contributed to generating ideas and writing the manuscript.
